# Parameter optimization of 3D convolutional neural network for dry-EEG motor imagery brain-machine interface

**DOI:** 10.3389/fnins.2025.1469244

**Published:** 2025-02-25

**Authors:** Nobuaki Kobayashi, Musashi Ino

**Affiliations:** Department of Precision Machinery Engineering, College of Science and Technology, Nihon University, Funabashi, Chiba, Japan

**Keywords:** nursing care, electroencephalography, motor imagery, brain-machine interface, brain-computer interface, convolutional neural network, edge computing

## Abstract

Easing the behavioral restrictions of those in need of care not only improves their own quality of life (QoL) but also reduces the burden on care workers and may help reduce the number of care workers in countries with declining birthrates. The brain-machine interface (BMI), in which appliances and machines are controlled only by brain activity, can be used in nursing care settings to alleviate behavioral restrictions and reduce stress for those in need of care. It is also expected to reduce the workload of care workers. In this study, we focused on motor imagery (MI) classification by deep-learning to construct a system that can identify MI obtained by electroencephalography (EEG) measurements with high accuracy and a low latency response. By completing the system on the edge, the privacy of personal MI data can be ensured, and the system is ubiquitous, which improves user convenience. On the other hand, however, the edge is limited by hardware resources, and the implementation of models with a huge number of parameters and high computational cost, such as deep-learning, on the edge is challenging. Therefore, by optimizing the MI measurement conditions and various parameters of the deep-learning model, we attempted to reduce the power consumption and improve the response latency of the system by minimizing the computational cost while maintaining high classification accuracy. In addition, we investigated the use of a 3-dimension convolutional neural network (3D CNN), which can retain spatial locality as a feature to further improve the classification accuracy. We propose a method to maintain a high classification accuracy while enabling processing on the edge by optimizing the size and number of kernels and the layer structure. Furthermore, to develop a practical BMI system, we introduced dry electrodes, which are more comfortable for daily use, and optimized the number of parameters and memory consumption size of the proposed model to maintain classification accuracy even with fewer electrodes, less recall time, and a lower sampling rate. Compared to EEGNet, the proposed 3D CNN reduces the number of parameters, the number of multiply-accumulates, and memory footprint by approximately 75.9%, 16.3%, and 12.5%, respectively, while maintaining the same level of classification accuracy with the conditions of eight electrodes, 3.5 seconds sample window size, and 125 Hz sampling rate in 4-class dry-EEG MI.

## 1 Introduction

The brain-machine interface (BMI) and brain-computer interface (BCI) are general terms for devices and systems that measure brain activity, analyze, and classify it using signal processing methods, and control devices according to the classification (Kobayashi and Nakagawa, 2015; Muller-Putz and Pfurtscheller, 2008; Ishizuka et al., 2020). Electroencephalography (EEG) (Tudor et al., 2005), intracranial electroencephalography (iEEG), and electrocorticography (ECoG) (Palmini, 2006; Komeiji et al., 2022) are commonly used to measure brain activity. Functional magnetic resonance imaging (fMRI) (Kamitani and Tong, 2005; Lauterbur, 1973), magnetoencephalography (MEG) (Hämäläinen et al., 1993), near infrared spectroscopy (NIRS) (Tsubone et al., 2007), positron emission tomography (PET) (van Elmpt et al., 2012), and computed tomography (CT) (Masuda et al., 2009), which use radioisotopes are also used. In this study, we focused on electrical measurements that have superior spatial and temporal resolutions. Electrical measurements of brain activity have been developed in two major ways: invasive and minimally invasive methods (Zippi et al., 2023), in which electrodes are implanted directly into brain neurons via craniotomy (ECoG or iEEG), and non-invasive methods (EEG), in which electrodes are placed on the scalp. In this study, we focused on the simple measurement of EEG, considering its ease of use in society, with the construction of a support system for nursing care. Although EEG is convenient and does not require surgery, it is susceptible to noise and has a lower signal-to-noise ratio (SN) than invasive methods because the electrodes are placed on the scalp. The SN is lower than that of invasive methods because it measures the attenuated electrical signals on the scalp after they pass through spinal fluid, meninges, and skull from the surface layer of the cerebral cortex. Therefore, the SN ratio is low, and the analysis of brain activity is much more difficult than with invasive methods. Therefore, non-invasive EEG contains various noises, and it is common to improve the SN by attenuating the noise using preprocessing, such as frequency filters. However, endogenous noise such as electrooculography (EOG) (Sugie and Jones, 1971) from eye movements and electromyography (EMG) (Fink and Scheiner, 1959) from body movements (these have larger amplitudes than EEG) and exogenous environmental noise such as commercial power supply and electromagnetic waves (these signals also have larger amplitudes than EEG) make it difficult to analyze the EEG. If the signal frequency, such as that of commercial power or electromagnetic waves, is known in advance, the SN can be improved by attenuating the components using a frequency filter (Meisler et al., 2019; Engin et al., 2007), as described above. Signal processing methods such as principal component analysis (PCA) (Xie and Krishnan, 2019), independent component analysis (ICA) (Katsumata et al., 2019), and empirical mode decomposition (EMD) (Samal and Hashmi, 2023) have been proposed to separate noise and EEG signals. Although these methods are effective in removing noise components to some extent, they are yet to completely identify and extract EEG components in advance. Therefore, as a countermeasure against noise, machine learning techniques have been used for EEG classification to map EEG signals onto the feature space and classify them to reduce the influence of noise and improve the accuracy.

Various types of EEG have been proposed for BMI, which can be classified into three main categories from the user's perspective: active, reactive, and passive. Active BMI is a type of EEG in which the user intentionally controls brain activity to operate a device, and is typically used when recalling a specific task. For example, motor imagery (MI) (Schloegl et al., 1997) is used to recall body movements and speech imagery is used to recall sound utterances. Reactive BMI is a method of presenting external stimuli such as visual stimuli to a user and extracting EEG features based on the user's responses. Typical examples of visual stimuli include the VEP system [CVEP (Riechmann et al., 2016) and SSVEP (Qin and Mei, 2018)] and event-related potentials (P300) (Chaurasiya et al., 2015) using an oddball task. The BCI speller uses this feature. Passive BMI reads the natural activity of the brain and aims to decode the user's intention, even if the user does not intend to. It would be ideal if this could be realized; however, at present, the information processing mechanism of the brain is not yet clear.

In this study, with the development of a BMI system for use in nursing care, we targeted active BMI, which is a simple measurement that can be used for a long time, to minimize the burden on the user and exclude stimulators from the system. Among active BMIs, we focused on MI, which has been gaining popularity in recent years because of its relatively easy recall and high classification accuracy. However, compared with reactive BMIs, active BMIs vary more than reactive BMIs in terms of recall and EEG features because the recall content and EEG features that emerge vary from person to person. Therefore, many attempts have been made to improve the accuracy of classification by training the recall in advance to reveal individual characteristics and by preparing materials (e.g., still or moving images) in advance to guide the user's recall, thereby making the EEG more versatile. Many attempts have been made to improve the classification accuracy by making EEG appear more versatile. In addition, MI has been attracting attention as a method of neurorehabilitation that improves the motor skills of stroke victims, and has a high affinity for this study as a BMI for use in nursing care. As mentioned above, machine learning is an essential technique for improving classification accuracy in noisy EEG measurements. Although various types of features such as event-related desynchronization and synchronization (ERD/ERS) (Rimbert et al., 2023) and common spatial pattern (CSP) (Belhadj et al., 2015) have been proposed for MI classification using conventional machine learning, it is difficult to select the features according to the task at hand. In recent years, deep-learning technology, which has made remarkable progress in image and natural language processing, has attracted attention, and its effectiveness has been demonstrated in many EEG classifications. Recurrent neural networks, such as long short-term memory (LSTM) (Hochreiter and Schmidhuber, 1997) and various MLP-type neural networks, such as EEGNet (Lawhern et al., 2018), TSFCNet (Zhi et al., 2023), MTFB-CNN (Li et al., 2023), MSHCNN (Tang et al., 2023), CMO-CNN (Liu K. et al., 2023), Incep-EEGNet (Riyad et al., 2020), EEGSym (Pérez-Velasco et al., 2022), EEG-TCNet (Ingolfsson et al., 2020), ETCNet (Qin et al., 2024), EEG-ITNet (Salami et al., 2022), TCNet-Fusion (Musallam et al., 2021), FB-Sinc-CSANet (Chen et al., 2023), SACNN-TFCSP (Zhang et al., 2023), and EISATC-Fusion (Liang et al., 2024), have enhanced motor MI classification accuracy through various strategies. These strategies include temporal convolution (Bai et al., 2018), fusion layers, residual blocks (He et al., 2016), and attention mechanisms, such as self-attention (Vaswani et al., 2017), etc.

To construct a BMI that can be easily used by people requiring care in a residential environment, it is necessary to satisfy the following three requirements: (1) wearability, (2) ultralow latency response, and (3) low power consumption. (1) is to have a small number of electrodes, minimize contact with the scalp, and ease the burden on the user owing to the prolonged use of dry electrodes that do not use materials such as conductive gels or pastes to keep contact impedance with the skin low. In addition, reducing the number of channels (#CHs) also means that the sensor mechanism of the system can be reduced, which reduces the number of dimensions of the required deep-learning model. Therefore, (3) is satisfied simultaneously. (2) can be solved using technology that provides processing power to the edge. (3) is related to (2). Although the cloud computing method consumes most of the power for data transfer (using the cloud, as well as other devices around us, requires the use of data centers with high power consumption and may cause network traffic if the number of devices increases, the edge can use EEG, which has the advantage of placing the sensor mechanism (analog front-end + A/D converter) and the processing mechanism (logic circuits) on the same device to provide high efficiency, comfort for the user, and confidentiality and privacy of data by completing the process on the local system. The advantages are that it is comfortable for the user and ensures data confidentiality and privacy by completing the process on the local system. However, the deep-learning model used to improve the identification accuracy of EEG requires a large amount of processing compared with conventional machine learning techniques and a large amount of memory because of the size of the network, making it difficult to achieve both (2) and (3). Considering the above, this paper makes the following contributions.

- Optimization of MI measurement conditions and deep-learning model parameters to reduce user burden and optimize hardware requirements (assuming that classification accuracy is maintained).
- Optimize the number of channels (#CHs) and electrode locations using the standard benchmark datasets: BCI Competition IV-2a (Tangermann et al., 2012) for 4-Class MI, and BCI Competition IV-2b (Tangermann et al., 2012) for 2-Class MI as the baseline.
- Optimize the number of input samples (sample windows) for MI to reduce the response latency of the system.
- Optimize the MI sampling frequency to reduce the power consumption of the analog-to-digital converter in the EEG sensor mechanism.
- The classification accuracy was compared between various deep-learning models and the proposed 3D-CNN (in this study, with-in subject as the training condition).

### 1.1 Related work

Both traditional machine learning and deep learning methods have been employed for EEG-based MI classification. In traditional machine learning, EEG features are typically extracted and fed into traditional classifiers, such as support vector machines (SVMs). Common spatial patterns (CSPs) (Belhadj et al., 2015) and their variants have also been widely used for feature extraction of MI. Filterbank CSPs (Ang et al., 2008) further enhance this process by dividing EEG signals into distinct frequency bands and applying CSPs to each band.

Most deep-learning approaches utilize raw EEG signals as input. Several prominent convolutional neural network (CNN)-based methods have demonstrated effectiveness. EEGNet (Lawhern et al., 2018) is a compact model designed to generalize across multiple EEG paradigms, while TSFCNet (Zhi et al., 2023) features a simple structure that minimizes overfitting. MTFB-CNN (Li et al., 2023) incorporates a multi-scale structure to extract high-level features across multiple scales, and MSHCNN (Tang et al., 2023) combines 1D and 2D convolutions for enhanced feature extraction. CMO-CNN (Liu et al., 2015) uses filters of varying scales and branch depths to extract diverse, multi-level features, while Incep-EEGNet (Riyad et al., 2020) employs an Inception layer with a compact parallel structure to efficiently extract multi-scale features. EEGSym (Pérez-Velasco et al., 2022) integrates an Inception module and a residual block, and EEG-TCNet (Ingolfsson et al., 2020) extracts high-level long-term dependencies by feeding temporal features generated by EEGNet into a TCN. ETCNet (Qin et al., 2024) combines efficient channel attention with TCN, and EEG-ITNet (Salami et al., 2022) incorporates an Inception layer and TCN for improved temporal modeling. TCNet-Fusion (Musallam et al., 2021) introduces a fusion layer to capture complex input data features and enhance the expressive power of EEG-TCNet. FB-Sinc-CSANet (Chen et al., 2023) employs channel self-attention to optimize local and global feature selection, SACNN-TFCSP (Zhang et al., 2023) integrates self-attention mechanisms, and EISATC-Fusion (Liang et al., 2024) combines self-attention with temporal depthwise separable convolution and a fusion layer. Lastly, Filterbank Multi-scale CNN (FBMSNet) (Liu X. et al., 2023) extends the concept of filterbanks from traditional machine learning to deep learning, enabling improved multi-scale feature extraction. Recently developed STMambaNet (Yang and Jia, 2024) captures long-range dependencies across both space and time while extracting detailed spatiotemporal dynamics of MI through selective state-space and quadratic self-attention mechanisms. As mentioned earlier, significant improvements in MI classification accuracy are often achieved by incorporating features that expand the feature values to higher dimensions and capture long-term dependencies. However, this typically requires additional layers and increases computational demands. Consequently, when considering deployment on edge devices, the key challenge lies in designing a hardware-efficient model that balances feature expansion to higher dimensions with the effective capture of long-term dependencies. To address this, MI-BMINet (Wang et al., 2024) has recently been introduced, offering reduced hardware resource requirements for edge implementation by optimizing parameters while maintaining high classification accuracy.

This study employs three-dimensional convolutional neural networks (3D-CNNs) to account for the spatial locality of EEG electrode mapping while scaling feature values to higher dimensions. In 2019, Zhao et al. introduced a multi-branch 3D-CNN (Zhao et al., 2019). A key advantage of 3D-CNNs lies in their ability to process input data with a three-dimensional structure, enabling feature extraction while preserving spatial information. In Zhao et al. (2019), three 3D-CNNs with varying receptive field sizes were implemented: the small receptive field network (SRF), medium receptive field network (MRF), and large receptive field network (LRF). These networks were arranged in a sequential chain, with outputs from each receptive field layer summed and passed to a softmax layer for final classification. Although this approach achieved exceptionally high classification accuracy, it involved a significant increase in parameters. Specifically, the 3D-CNNs improved classification accuracy by 2.20% to 3.71% on average but required 12.67 k to 326.42 k times more parameters and 2.81 to 67.11 times more #MACCs compared to EEGNet (Lawhern et al., 2018). Beyond MI, the application of 3D features has also proven effective in other domains. For example, MetaEmotionNet (Ning et al., 2024) leverages 3D spatial-spectral information in a spatial-spectral-temporal-based attention 3D dense layer (3D attention mechanism) to accurately classify four emotional states—neutral, fear, sadness, and happiness.

Increasing the number of layers and adding functionalities to deep neural networks inevitably increase computational complexity, limiting their applicability on edge devices. This study explored the use of a 3D-CNN that preserves spatial locality to enhance classification accuracy. However, the introduction of 3D convolutional layers also expands feature dimensions, leading to a significant increase in parameters and computational costs. To address this, the computational burden was minimized by optimizing various parameters, enabling efficient edge processing while maintaining high classification accuracy. Section 3 details a proposed method for mitigating the increase in the number of parameters (#parameters) and multiply-accumulates (#MACCs) using the Conv. 3D layer, improving classification accuracy and optimizing the network structure.

## 2 Establishment of optimal conditions for 4-Class and 2-Class motor imagery measurement to reduce user burden and minimize hardware requirements

To estimate the minimum system performance requirements to maintain classification accuracy in a BMI system using 4-Class and 2-Class motor imagery (MI), this section uses the BCI Competition IV-2a Dataset (BCI-IV2a) (Tangermann et al., 2012), BCI Competition IV-2b Dataset (BCI-IV2b), and deep-learning models, including EEGNet (Lawhern et al., 2018), EEG-TCNet (Ingolfsson et al., 2020), TCNet-Fusion (Musallam et al., 2021), and EISATC-Fusion (Liang et al., 2024), to optimize various parameters for 4-Class and 2-Class MI measurement. The primary objective of this study is to develop a compact model with high classification accuracy. Therefore, four models were selected—those capable of achieving high classification accuracy with a relatively small number of parameters (#parameters) and those incorporating schemes to improve classification accuracy. The impact of parameter changes in MI measurement on the classification accuracy of each model was then observed. Specifically, we estimated the optimal values for the channel (CH) selection, sample window size, and sampling rate. Sections 2.1.1 to 2.1.3 outline the significance and purpose of optimizing each parameter, and Sections 2.2.1 to 2.2.6 describe the experimental conditions necessary for each optimization.

### 2.1 4-Class and 2-Class motor imagery measurement condition parameter optimization based on classification accuracy

#### 2.1.1 Manual channel selection

**Figure 1:**
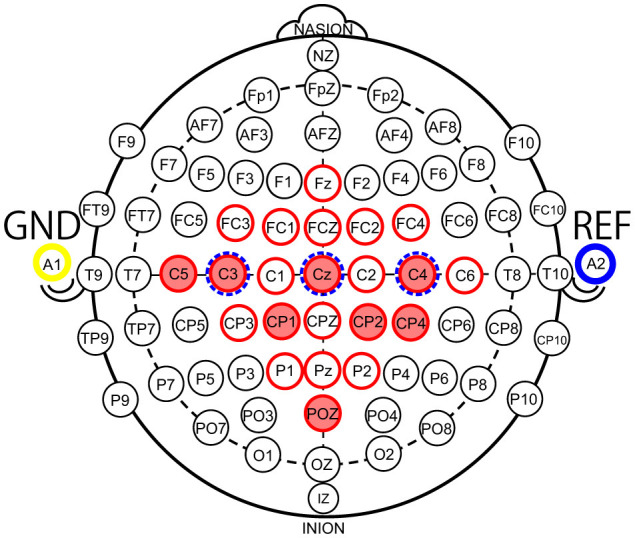
Electrode locations according to the 10–10 international system (red circles: electrode locations of 22 channels used in the BCI Competition IV-2a Dataset, red fill: electrode locations of eight channels used in this study, blue dotted circles: electrode locations of 3 channels used in the BCI Competition IV-2b Dataset).

Reducing the number of channels (#CHs) is effective in simplifying the BMI system, and reducing the number of electrodes placed on the scalp when measuring MI can reduce the burden on the user when wearing the system and the cost of the system. In a typical EEG-based BMI system (Liu et al., 2015), voltage signals measured by electrodes on the scalp are amplified by an amplifier (amp), converted to digital values by an analog-to-digital converter (A/D converter), and then classified by a processor. Therefore, reducing the number of electrodes reduces the cost of the electrodes, amp, and A/D converter circuits, which means that the size and power consumption of the analog front-end circuit can be reduced. In addition, the number of input dimensions in the machine-learning model is reduced, which reduces the computational cost of the processor so that the power consumption of the digital processor can be reduced as well. However, the optimal #CHs and scalp locations for MI classification may differ from one user to another. Therefore, useful algorithms have been proposed to select these automatically for each user (Wang et al., 2024). However, to narrow down the optimal #CHs and electrode locations for each user, it is necessary to conduct motor imagery acquisition experiments with a large number of electrodes prepared in advance, and to determine the optimal #CHs and electrode locations based on the analysis results of the acquired MI according to an algorithm, or so-called calibration. To promote the use of BMI, it is important to design a user interface that is calibration-less and less difficult to use. Therefore, we identified the minimum #CHs and electrode locations that could maintain the average classification accuracy for all nine subjects using BCI-IV2a and BCI-IV2b. The BCI-IV2a is the MI of nine subjects obtained with 22 electrode CHs (red circles) installed in the configuration shown in Figure 1, and BCI-IV2b is the MI of nine subjects obtained with 3 electrode CHs (blue dotted circles), as illustrated in Figure 1.

#### 2.1.2 Sample window size

An electroencephalography (EEG) is a time-series voltage signal. In MI, the user is asked to recall a target body movement for a certain period, and the MI during the recall is used to obtain the features. However, the longer the recall time, the greater the load on the user; thus, there is a tradeoff between classification accuracy and user load in terms of recall time. In the case of deep-learning, as the recall time decreases, the number of MI data samples decreases, and the number of MI data dimensions input to the deep-learning model is also reduced, thus reducing the circuit and computational costs. Therefore, we optimized the sample window size in the MI time direction. Specifically, we seek a minimum sample window size that satisfies the requirement of maintaining high classification accuracy in the recall time per trial of BCI-IV2a and BCI-IV2b. The recall time required to maintain classification accuracy (the time required to obtain salient features) is expected to vary widely among individuals; therefore, the objective here is to estimate the recall time that can maintain high classification accuracy on average.

#### 2.1.3 Downsampling

As mentioned previously, the EEG-based BMI system measures brain activity as analog voltage values in the sensor section (analog front-end). The measured analog voltage values were converted to digital values using an A/D converter and processed using deep-learning to handle discrete values. Lowering the sampling rate reduces the charge-discharge rate per unit time of MOSFETs in CMOS circuits and the oscillation frequency of the oscillator circuit, such as a phase-locked loop (PLL) for sampling, thus reducing the power consumption of the analog front-end. In addition, a decrease in the sampling rate reduces the number of data samples, which, as previously mentioned, reduces the number of input dimensions for the deep-learning model. Because the sample frequency of MI in BCI-IV2a and BCI-IV2b was 250 Hz, we downsampled this data by a factor of 1/*n* (*n* = 1, 2, 3, 4, 5) (1:*n* subsamples) and observed the effect on the accuracy by classifying the data using the deep-learning model.

### 2.2 Experiments aimed at optimizing measurement conditions

#### 2.2.1 Configuration and experimental procedures

Table 1 lists the experimental environments and conditions used for the optimization of the channel selection, sample window size, and sampling rate. First, the following procedure was used to optimize the manual channel selection.

- From the *n* channels (CHs), *r* (*r* = *n*-2) are extracted and 5-fold cross validation is performed on each channel (e.g., for *n* = 22, the number of combinations is _22_C_21_ = 231).
- The 10 combinations with the highest accuracy and the 10 combinations with the lowest accuracy are extracted from each average classification accuracy.
- The number of times a channel appears in the extracted combinations is used to identify the CHs with low usefulness.
- If the extracted counts (frequency counts) are equal, the selection of CHs is expanded to 15.
- If the number of CHs (#CHs) is also equal, we select the CHs to be deleted by learning CHs with the highest accuracy.
- This is performed for 22 CHs up to 10 CHs.
- The following procedure is used to determine the minimum #CHs that can be removed while maintaining accuracy.
- After 10 CHs, select one channel at a time.
- Select nine out of 10 CHs and perform 5-fold cross validation twice (_10_C_9_ × 2 = 32).
- The combination with high accuracy is identified based on the above-average classification accuracy.
- Conduct 5-fold cross validation 10 times under the same conditions using the identified channel combinations.
- Repeat this process to identify the minimum #CHs for which accuracy can be maintained.
- For BCI-IV2b, as this dataset was obtained using three CHs, we performed all combinations of two CHs (_3_C_2_ = 6) and each individual CH among the three (_3_C_1_ = 3).

**Table 1:** Experimental environment.

**Parameter**	**Value (version, quantity, type, etc.)**
Python	3.8.18
Pytorch	1.13.1
GPU	NVIDIA GeForce RTX 3090
CUDA	11.6
DATASET	BCI Competition IV-2a Dataset (Tangermann et al., 2012): (It includes motor imagery measured by 9 subjects who placed 22 electrodes on the location indicated as red circles in Figure 1) BCI Competition IV-2b Dataset (Tangermann et al., 2012): (It includes motor imagery measured by 9 subjects who placed 3 electrodes on the location indicated as blue dotted circles in Figure 1)
Preprocess	Bandpass filter: 0.5–60 Hz, Notch filter: 48–52 Hz Normalization using StandardScaler in Scikit-Learn (Removing mean and scaling to unit-variance per channel, based on the statistics of the training set)
Manual channel selection (Each location is denoted in Table 4: The basis for selecting the electrode location is shown in Section 2.2.3)	(BCI-IV2a) #channels [CH]: 22, 20, 18, 16, 14, 12, 10, 9, 8, 7, 6, 5, 4, 3, 2, 1 (BCI-IV2b) #channels [CH]: 3, 2, 1
Sample window size [s]	4, 3.5, 3, 2.5, 2, 1.5, 1, 0.5 (0.5 s increments) Use only one sample window, with the start of the sample window at *t* = 2 (BCI-IV2a, BCI-IV2b) [s] for each trial (when each cue is indicated).
Sampling rate (ratio of down-sampling: 250 Hz as reference 1) [Hz]	250 (1/1), 125 (1/2), 62.5 (1/3), 31.25 (1/4), 15.625 (1/5) (number of subsamples *n* = 1, 2, 3, 4, 5)
Models	EEGNet (Lawhern et al., 2018), EEG-TCNet (Ingolfsson et al., 2020), TCNet-Fusion (Musallam et al., 2021), and EISATC-Fusion (Liang et al., 2024)
Model parameter	default settings in Lawhern et al. (2018), Ingolfsson et al. (2020), Musallam et al. (2021), and Liang et al. (2024)
Epoch (early stop epoch)	3,000 (300)
Batch size	64
Training Method	Within-subject (subject-specific) (BCI-IV2a) 288 trials of first session is used for training [80% (230 trials) for training, 20% (58 trials) for validation], 288 trials of second session is used for testing (BCI-IV2b) 400 trials are used for training [80% (320 trials) for training, 20% (80 trials) for validation] and 320 trials are used for testing. (Both BCI-IV2a and BCI-IV2b) The number of samples is assumed to be the same for training, validation, and testing, depending on the sample window size. The random seed for shuffling is the same throughout the training.
Optimizer	Adam
Loss function	Cross entropy loss
Verification methodology	5-fold cross validation

The total recall time, including the cues, was 4 seconds (s) for BCI-IV2a and BCI-IV2b. Therefore, in this experiment, the maximum sample window size was 4 s, and the sample window size was decreased in increments of 0.5 s to a minimum of 0.5 s. In all cases, the starting point was *t* = 2 s at the beginning of the cue, and one sample window was used per trial (to standardize the number of training, validation, and test samples for all training sessions). Finally, for downsampling, because the sampling rate of MI data in BCI-IV2a and BCI-IV2b is 250 Hz, we used 250 Hz as reference (1) and applied downsampling (1:*n* subsampling) to 1/*n* (*n* = 1, 2, 3, 4, 5) from this point to obtain five different time samples, *T*. The classification accuracy for each of the five types was calculated and the optimal value was obtained. The results of the experiments are described in the following sections. To maintain the training data under the same conditions for the 5-fold cross validation, the random seeds for shuffling were standardized.

#### 2.2.2 Optimization of measurement condition parameters among deep-learning models

The optimization of measurement condition parameters was based on the classification accuracy of EEGNet (Lawhern et al., 2018), EEG-TCNet (Ingolfsson et al., 2020), TCNet-Fusion (Musallam et al., 2021), and EISATC-Fusion (Liang et al., 2024), which served as standard benchmarks for MI classification. The measurement condition parameters, along with the training parameters (hyperparameters) for each model, were set to the default values used in the respective references. In this experiment, we used a within-subject (subject-specific) model, rather than a cross-subject (subject-independent) or fine-tuned model. The classification accuracies of these four models with their default settings, before optimizing the measurement conditions, are shown in Table 2 (BCI-IV2a) and Table 3 (BCI-IV2b). Table 2 shows that the mean classification accuracies of the nine subjects in BCI-IV2a (22 channels, 4 s, subsample *n* = 1) were highest for EISATC-Fusion, followed by EEG-TCNet, TCNet-Fusion, and EEGNet. The statistical significance is analyzed using the Wilcoxon signed-rank test between EEGNet and the other models each (*p* < 0.01). EISATC-Fusion achieved the best standard deviation (std. dev.) and kappa score, indicating it is a stable model with low statistical variability. This suggests that the various schemes incorporated in EISATC-Fusion, such as Inception, Attention, and Temporal Convolutional Network (TCN), are effective. In contrast, the number of parameters (#parameters) is largest for EISATC-Fusion, followed by TCNet-Fusion, EEG-TCNet, and EEGNet. EISATC-Fusion has 82.62 times more parameters than EEGNet, the model with the fewest parameters. The number of multiply-accumulates (#MACCs) for EISATC-Fusion was 2.494 times larger than that of EEGNet. EISATC-Fusion's memory footprint was 4.223 times larger than EEGNet's. In contrast, EEG-TCNet's #MACCs was about half that of EEGNet, with a slight increase in #parameters (1.175 times larger than EEGNet). Additionally, memory footprint of EEG-TCNet remained comparable to that of EEGNet. While TCNet-Fusion achieved the same classification accuracy as EEG-TCNet, its #parameters, #MACCs, and memory footprint were 4.743, 1.569, and 2.914 times larger than those of EEGNet, respectively. Despite having the largest number of parameters, EISATC-Fusion stands out for its classification accuracy and stability. In contrast, EEGNet, with the smallest number of parameters and the third-highest classification accuracy, and EEG-TCNet, which has the second-highest classification accuracy, the second-smallest #MACCs, and the smallest memory footprint, offer high cost-performance, even though their classification accuracies are lower than that of EISATC-Fusion. In BCI-IV2b (3 CHs, 4 s, subsample *n* = 1), shown in Table 3, the number of channels and classes is smaller than in BCI-IV2a, and there is marginal difference in classification accuracy between the models (EISATC-Fusion is up to 1.92% higher than EEGNet). However, in terms of model size (#parameters, #MACCs, and memory footprint), the trend is similar to that of BCI-IV2a, though the scales differ. Using these values as benchmarks, we confirm the effects of optimizing the measurement and model parameters in the following sections.

**Table 2:** Baseline comparison among four models for the 4-Class BCI-IV2a.

**Model**		**EEGNet (Lawhern et al., 2018)**	**EEG-TCNet (Ingolfsson et al., 2020)**	**TCNet-Fusion (Musallam et al., 2021)**	**EISATC-Fusion (Liang et al., 2024)**
#channels [CH]		22	22	22	22
Sample window size [s]		4.0	4.0	4.0	4.0
Subsample, *n*		1	1	1	1
Accuracy	Sub1	0.8556	0.8493	0.8611	**0.8951**
	Sub2	0.6500	0.6645	0.6708	**0.7188**
	Sub3	0.9083	0.9236	0.9271	**0.9597**
	Sub4	0.6069	0.7389	0.6854	**0.8020**
	Sub5	0.7347	**0.7903**	0.7694	0.7896
	Sub6	0.5979	**0.6611**	**0.6611**	0.6389
	Sub7	0.8938	0.9194	0.9181	**0.9306**
	Sub8	0.8236	0.8361	**0.8611**	0.8493
	Sub9	0.7903	0.8583	0.8229	**0.9063**
	Mean	0.7623	0.8046	0.7975	**0.8322**
	(Difference: EEGNet = 0)	(0.000)	(+0.0423)	(+0.0352)	**(+0.0699)**
	Std. dev.	0.02568	0.01790	0.02089	**0.01499**
	p-value	-	1.81e–05	1.17e–04	**5.01e–08**
Kappa score		0.6878	0.7450	0.7222	**0.7680**
#parameters [k]		**3.444**	4.048	16.336	284.553
(Ratio: EEGNet = 1)		**(1.000)**	(1.175)	(4.743)	(82.62)
#MACCs [M]		11.75	**6.10**	18.43	29.30
(Ratio: EEGNet = 1)		(1.000)	**(0.519)**	(1.569)	(2.494)
Memory footprint [MB]		3.27	**3.24**	9.53	13.81
(Ratio: EEGNet = 1)		(1.000)	**(0.991)**	(2.914)	(4.223)

The best values are highlighted in bold.

**Table 3:** Baseline comparison among four models for the 2-Class BCI-IV2b.

**Model**		**EEGNet (Lawhern et al., 2018)**	**EEG-TCNet (Ingolfsson et al., 2020)**	**TCNet-Fusion (Musallam et al., 2021)**	**EISATC-Fusion (Liang et al., 2024)**
#channels [CH]		3	3	3	3
Sample window size [s]		4.0	4.0	4.0	4.0
Subsample, *n*		1	1	1	1
Accuracy	Sub1	0.7713	0.7556	0.7656	**0.7856**
	Sub2	0.7250	0.7386	0.7300	**0.7436**
	Sub3	**0.8906**	0.8806	0.8825	0.8694
	Sub4	**0.9813**	**0.9813**	0.9769	0.9788
	Sub5	0.9544	0.9625	0.9706	**0.9769**
	Sub6	0.8563	0.8413	0.8888	**0.8956**
	Sub7	0.9231	0.9200	0.9206	**0.9268**
	Sub8	**0.9544**	0.9350	0.9393	0.9462
	Sub9	0.7763	0.8600	0.8719	0.8831
	Mean	0.8703	0.8750	0.8829	**0.8895**
	(Difference: EEGNet = 0)	(0.000)	(+0.0047)	(+0.0126)	**(+0.0192)**
	Std. dev.	0.02561	0.01123	0.00853	**0.00841**
	*p*-value	-	4.35e–02	7.07e–02	**2.93e–02**
Kappa score		0.7389	0.7516	0.7658	**0.7695**
#parameters [k]		**2.146**	3.718	12.182	281.517
(Ratio: EEGNet = 1)		**(1.000)**	(1.733)	(5.677)	(131.2)
#MACCs [M]		1.71	**0.93**	2.92	8.62
(Ratio: EEGNet = 1)		(1.000)	**(0.544)**	(1.708)	(5.041)
Memory footprint [MB]		0.76	**0.73**	2.14	4.00
(Ratio: EEGNet = 1)		(1.000)	**(0.961)**	(2.816)	(5.263)

The best values are highlighted in bold.

#### 2.2.3 Experimental results on manual channel selection

**Figure 2:**
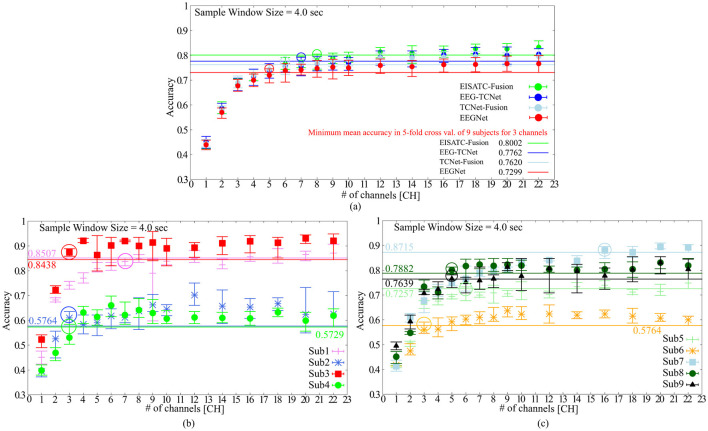
The classification accuracy vs. the number of channels **(A)** 5 deep-learning models, **(B)** per subject: Sub1–Sub4 for EEGNet, **(C)** per subject: Sub5–Sub9 for EEGNet.

Table 4 lists the optimal electrode locations corresponding to the #CHs selected based on the experimental conditions and procedures described in Section 2.2.1. Figure 2A shows the relationship between the mean classification accuracy and CHs for the four deep-learning models trained using the MI of the CHs listed in this table, and (Figure 2B) four subjects (Sub1 to Sub4) and (Figure 2C) five subjects (Sub5 to Sub9) show the results for each subject on EEGNet (the result of EEGNet is only shown to improve visibility). The graphs show the minimum value (lower limit of the error bar), maximum value (upper limit of the error bar), and mean value (filled circle) of the values obtained in the 5-fold cross validation. The minimum classification accuracy obtained for a sample window size of 22 CHs and 4.0 s is indicated by the solid line. The minimum classification accuracy of the nine subjects for EEGNet is 0.7299, whereas the accuracies of each subject are 0.8507 (Sub1), 0.5764 (Sub2), 0.8438 (Sub3), 0.5729 (Sub4), 0.7257 (Sub5), 0.5764 (Sub6), 0.8715 (Sub7), 0.7882 (Sub8), and 0.7639 (Sub9). Additionally, the minimum classification accuracies of the other models were 0.8002 (EISATC-Fusion), 0.7762 (EEG-TCNet), and 0.7620 (TCNet-Fusion), respectively.

**Table 4:** Electrode locations selected by manual channel selection.

**#channels [CH]**	**Optimal electrode location for each number of channels**
22	FZ, FC3, FC1, FCZ, FC2, FC4, C5, C3, C1, CZ, C2, C4, C6, CP3, CP1, CPZ, CP2, CP4, P1, PZ, P2, POZ
20	FZ, FC3, FC1, FCZ, FC2, FC4, C5, C3, CZ, C2, C4, CP3, CP1, CPZ, CP2, CP4, P1, PZ, P2, POZ (remove C1, C6 from 22 CHs)
18	FZ, FC3, FC1, FCZ, FC2, C5, C3, CZ, C2, C4, CP3, CP1, CPZ, CP2, CP4, P1, P2, POZ (remove FC4 and PZ from 20 CHs)
16	FZ, FC3, FC1, FCZ, C5, C3, CZ, C2, C4, CP3, CP1, CPZ, CP2, CP4, P2, POZ (remove FC2, P1 from 18 CHs)
14	FZ, FC1, FCZ, C5, C3, CZ, C4, CP3, CP1, CPZ, CP2, CP4, P2, POZ (remove FC3, C2 from 16 CHs)
12	FC1, C5, C3, CZ, C4, CP3, CP1, CPZ, CP2, CP4, P2, POZ (Remove FZ, FCZ from 14 CHs)
10	FC1, C5, C3, CZ, C4, CP1, CPZ, CP2, CP4, POZ (Remove CP3 and P2 from 12 CHs)
9	C5, C3, CZ, C4, CP1, CPZ, CP2, CP4, POZ (remove FC1 from 10 CHs)
**8**	**C5, C3, CZ, C4, CP1, CP2, CP4, POZ (corresponds to red-filled locations in** **Figure 1****)** (remove CPZ from 9 CHs)
7	C5, C3, CZ, C4, CP1, CP2, POZ (Delete CP4 from 8 CHs)
6	C3, CZ, C4, CP1, CP2, POZ (remove C5 from 7 CHs)
5	C3, CZ, C4, CP1, POZ (remove CP2 from 6 CHs)
4	C3, CZ, C4, POZ (remove CP1 from 5 CHs)
3	C3, CZ, C4 (remove POZ from 4 CHs)
2	C3, C4 (CZ removed from 3 CHs)
1	C4 (C3 removed from 2 CHs)

The selected eight channels in this study are highlighted in bold.

As mentioned above, the objective of this experiment was to determine the minimum #CHs that could maintain classification accuracy for all nine subjects. The required #CHs varied depending on how “maintaining classification accuracy” is defined. Here it is defined as the maximum classification accuracy obtained by 5-fold cross validation which exceeds the minimum classification accuracy obtained by 5-fold cross validation using the largest 22 CHs. On EEGnet, the minimum #CHs for each subject were seven CHs (Sub1), three CHs (Sub2), three CHs (Sub3), and three CHs (Sub4) from Figure 2B, six CHs (Sub5), three CHs (Sub6), six CHs (Sub7), five CHs (Sub8), and five CHs (Sub9), as shown in Figure 2C. (All values are indicated by colored in the graph). In other words, the CHs required to maintain classification accuracy varied from subject to subject, with a median of five and a mean of approximately 5.667 for all nine subjects on EEGNet. From the above, we set the minimum CHs required to maintain classification accuracy at five (only Sub7 deviated significantly from the median and mean; therefore, it is treated as an outlier here) on EEGNet. Furthermore, as illustrated in Figure 2A, the #CHs that could maintain the classification accuracy for all nine subjects was above five (EEGNet), eight (EISATC-Fusion), seven (EEG-TCNet), and six (TCNet-Fusion) CHs, respectively. In this study, we used eight CHs as the optimal values in the demonstration experiments from Section 4 onward. The optimal electrode locations in this experiment were C5, C3, CZ, C4, CP1, CP2, CP4, and POZ (corresponding to the red-filled locations in Figure 1), as shown in Figure 2 in bold.

#### 2.2.4 Experimental results on sample window size

**Figure 3:**
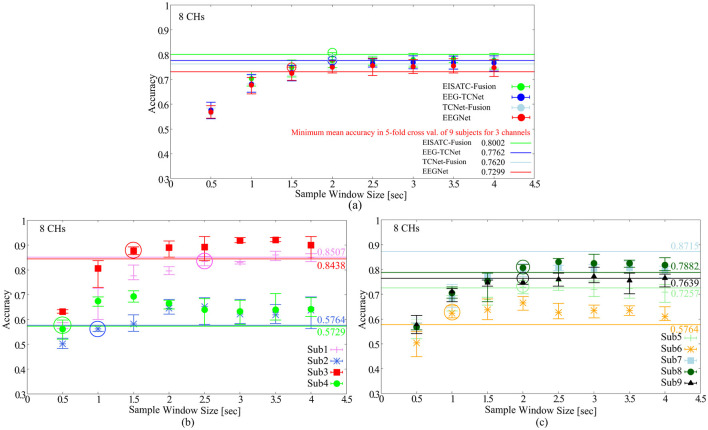
Classification accuracy vs. sample window size **(A)** 5 deep-learning models, **(B)** per subject: Sub1–Sub4 for EEGNet, **(C)** per subject: Sub5–Sub9 for EEGNet.

As mentioned earlier, the objectives of this experiment were to minimize the recall time and reduce the number of input dimensions for the deep-learning model. Here, we determined the minimum recall time that can maintain the classification accuracy for all nine subjects. The definition of “maintaining classification accuracy” is the same as in the previous section. The #CHs used in the analysis is the eight CHs as in the previous section. Figure 3A shows the relationship between the mean classification accuracy and sample window size for the four deep-learning models, (Figure 3B) four subjects (Sub1 to Sub4), and (Figure 3C) five subjects (Sub5 to Sub9) show the results for each subject (the result of EEGNet is only shown to improve visibility). The contents of these graphs are identical to those described in the previous section. The comparison targets are the minimum classification accuracy (solid line) obtained by 5-fold cross validation with the maximum #CHs (22) at the maximum sample window size (4.0 s).

**Figure 4:**
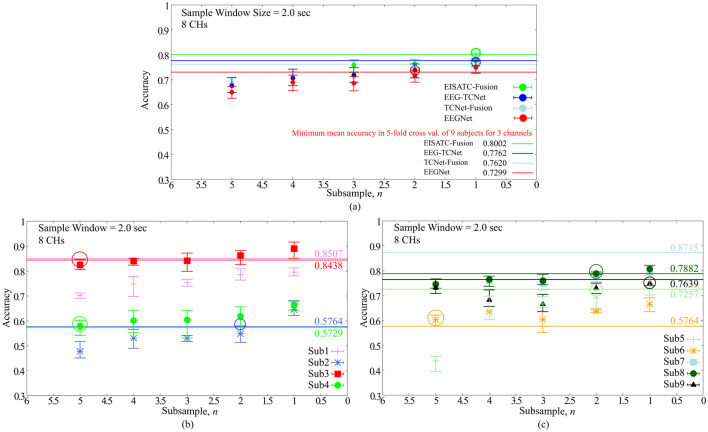
Classification accuracy vs. number of subsamples **(A)** 5 deep-learning models, **(B)** per subject: Sub1–Sub4 for EEGNet, **(C)** per subject: Sub5–Sub9 for EEGNet.

Figure 3A shows that the sample window size that can maintain the classification accuracy is between 2.0 s and 1.5 s among the four deep-learning models. By subject, Figure 3B shows 2.5 s (Sub1), 1.0 s (Sub2), 1.5 s (Sub3), 0.5 s (Sub4), Figure 4C shows 2.0 s (Sub5), 1.0 s (Sub6), 2.0 s (Sub6), 2.0 s (Sub8), 2.0 s (Sub9) are the minimum values required to maintain the classification accuracy (all indicated by colored circles in the graph). In other words, the sample window size required to maintain classification accuracy varied from subject to subject, and the median and mean values for all eight subjects (excluding Sub7, which is an outlier) were 1.75 and 1.563, respectively. EEGNet, EISATC-Fusion, EEG-TCNet, and TCNet-Fusion are illustrated in Figure 3A. The sample window size required to maintain classification accuracy for all nine subjects was above 1.5 s for EEGNet, and above 2.0 s for EISATC-Fusion, EEG-TCNet, and TCNet-Fusion. Based on the above, the minimum sample window size required to maintain classification accuracy was set to 2.0 s.

#### 2.2.5 Experimental results on downsampling

Finally, the optimal value of the sampling frequency is obtained by downsampling. As mentioned earlier, the purpose of this experiment was to reduce the sampling frequency to increase the power efficiency of the EEG sensor (analog front-end) and reduce the number of input dimensions for deep-learning models. As in the previous section, we determined the minimum sampling frequency that could maintain the classification accuracy for all nine subjects. Two hundred and fifty Hz is base 1, as BCI-IV2a is an MI acquired with a sampling frequency of 250 Hz (sps) and *n* times the sampling interval (one point per *n* data points, as we refer to *n* as the number of sub-samplings). The definition of “maintenance of classification accuracy” is the same as previous sections. The CHs and sample window size were eight CHs and 2.0 s, respectively, as described in the previous section. Figure 4A shows the relationship between the mean classification accuracy and sample window size for the four deep-learning models, (Figure 4B) 4 subjects (Sub1 to Sub4), and (Figure 4C) five subjects (Sub5 to Sub9) show the results for each subject. The contents of these graphs are identical to those described in the previous sections. The comparison targets are the minimum classification accuracy (solid line) obtained by 5-fold cross validation with the maximum #CHs (22) and maximum sample window size (4.0 s).

Figure 4A shows that the mean number of subsamples (#subsamples) that can maintain the classification accuracy is 1 and 2 for the four deep-learning models, and we conclude that 2 is the maximum number (indicated by the colored circles in the graph). By subject, Figure 4B shows that Sub1 (outlier as shown in the previous section), 2 (Sub2), 5 (Sub3), and 5 (Sub4), and Figure 4C shows that 2 (Sub5), 5 (Sub6), Sub7 (outlier as shown in the previous section), 2 (Sub8), and 1 (Sub9) are the maximum number (all indicated by the colored circles in the graph). In other words, the #subsamples required to maintain the classification accuracy varied from subject to subject; the median value for all seven subjects (excluding Sub1 and Sub7, which were outliers) was 2.00, and the mean value was approximately 3.14. From the above, we set the maximum #subsamples necessary to maintain classification accuracy to 2. It should be noted that although a certain degree of degradation of classification accuracy is unavoidable, if this is tolerated, the sampling frequency can be reduced in proportion to the number of subsamples, which is expected to be highly effective from the standpoint of the circuit, particularly in terms of analog front-end circuit power efficiency.

#### 2.2.6 2-Class and 3-CHs BCI-IV2b

**Figure 5:**
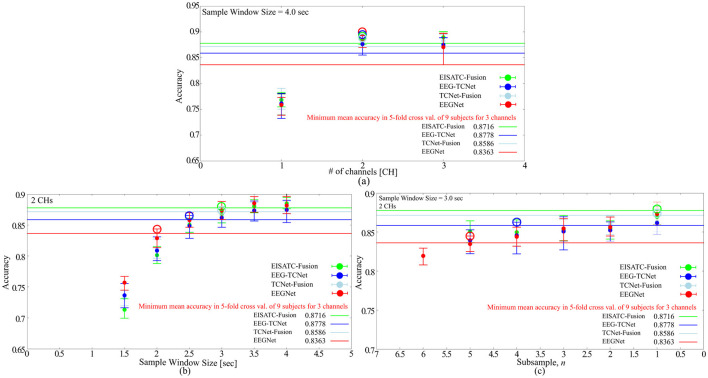
Optimization of measurement condition parameters with BCI-IV2b for 5 deep-learning models **(A)** number of channels, **(B)** sample window size, **(C)** subsample *n*.

In the previous sections, we discussed the optimization of the measurement condition parameters for BCI-IV2a. However, we also examined the trend for BCI-IV2b, which has fewer classes and #CHs. Figure 5 illustrates the relationship between (Figure 5A) #CHs, (Figure 5B) sample window size, and (Figure 5C) subsample *n*, each obtained by training BCI-IV2b with the four deep-learning models, and the mean classification accuracy for nine subjects. The optimal condition parameters for #CHs, sample window size, and subsample *n* were 2 CHs, 3.0 s, and 1, respectively, when the same method used for BCI-IV2a was applied to obtain the optimal parameters.

## 3 Utilization of 3D convolutional neural network

### 3.1 Proposed 3D-CNN model

The purpose of the proposed 3D-CNN is to improve classification accuracy by including spatial information in the input data and simultaneously reducing the model size. The structure of the proposed model is presented in Table 5. The model structure was inspired by EEGNet by combining the Conv. 2D and Depthwise Conv. 2D, which extracts the first frequency and channel components of the EEGNet into a single layer of Conv. 3D to reduce the model size, computational complexity, and memory 3D into a single layer, thus reducing the size of the model, amount of computation, and memory footprint. To reduce the increase in the number of parameters (#parameters), the proposed model simultaneously incorporates frequency and location information in the first Conv. 3D layer, and estimates additional features from this information in the next Separable Conv. 3D layer. Finally, the features converged in the dense layer and were classified using a softmax function. The meaning of each model parameter is listed in Table 5. *T* is the time sample {sample window size [s] × 250 [Hz] (sampling frequency of the acquired MI) × (1/number of subsamples, *n*)}. In addition, a coefficient *K*_s_ is provided to extend the number of dimensions of the kernel in Separable Conv3D so that more detailed features can be output by increasing this value.

**Table 5:** Structure of the proposed 3D-CNN.

**Block**	**Layer**	**#Filters**	**Size**	**Output**	**Options**
1	Input			(*C*_1_, *C*_2_*, T*)	*T:* Number of time samples *C*_1_: row direction *C*_2_: column direction
	Reshape			(1, *C*_1_, *C*_2_*, T*)	
	Conv3D	*F*_1_	(*C*_1_, *C*_2_, *K*_l_*)*	(*D* ^*^F_1_, 1, 1, *T*)	*K*_l_: Kernel length *F*_1_: Number of temporal filters *D*: Depth
	BatchNorm			(*D* ^*^F_1_, 1, 1, *T*)	
	Activation			(*D* ^*^F_1_, 1, 1, *T*)	ELU
	MaxPool3D		(1, 1, 4)	(*D* ^*^F_1_, 1, 1, *T/*/8)	
	Dropout			(*D* ^*^F_1_, 1, 1, *T/*/8)	Dropout rate = 0.5
2	Separable Conv3D	*F*_2_	(*K*_s_, *K*_s_, *C*_1_^*^ *C*_2_^*^ *K*_s_)	(*F*_2_, 1, 1, *T/*/8)	*F*_2_: Number of spatial filters *K*_s_: Kernel extension coefficients
	BatchNorm			(*F*_2_, 1, 1, *T/*/8)	
	Activation			(*F*_2_, 1, 1, *T/*/8)	ELU
	MaxPool3D		(1, 1, 8)	(*F*_2_, 1, 1, *T/*/64)	
	Dropout			(*F*_2_, 1, 1, *T/*/64)	Dropout rate = 0.5
3	Flatten			(*F*_2_ ^*^ T//64)	
Classifier	Dense			*N*	Softmax, *N* (Number of Classes) = 4

### 3.2 Effect of input data shape on classification accuracy of proposed 3D-CNN

**Figure 6:**
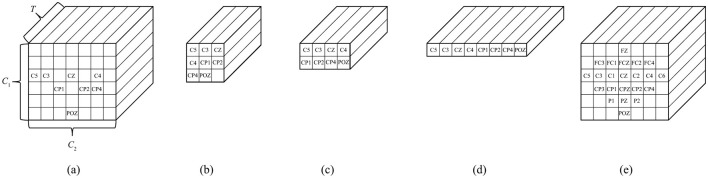
Shapes of input motor imagery data to the proposed 3D-CNN **(A)** 8 CHs, *C*_1_ = 6, *C*_2_ = 7, **(B)** 8 CHs, *C*_1_ = 3, *C*_2_ = 3, **(C)** 8 CHs, *C*_1_ = 2, *C*_2_ = 4, **(D)** 8 CHs, *C*_1_ = 1, *C*_2_ = 8, **(E)** 22 CHs, *C*_1_ = 6, *C*_2_ = 7.

As mentioned in Section 3.1, the proposed 3D-CNN provides the location information of the EEG channels in the input form to preserve spatial features. The #CHs required to maintain this classification accuracy was eight (shown in red in Figure 1), as described in Section 2.2.3. The electrode locations of the eight CHs (C5, C3, CZ, C2, CP1, CP2, CP4, and POZ) were not necessarily adjacent to each other vertically, horizontally, and laterally, and the #CHs required in the line direction from the NASION side in Figure 1, the first four rows (C5, C3, CZ, C2), the second three rows (CP1, CP2, CP4), and one row in the third row (POZ) were counted. The channel arrangement that can cover these elements is expressed as an array, for example, three rows and four columns would have 12 elements, resulting in four unnecessary elements of the required eight elements (eight CHs). To maintain as much spatial information as possible and improve classification accuracy, the existence of unnecessary elements must be tolerated; however, from a computing perspective, unnecessary calculations should be avoided as much as possible. Therefore, we used the five 3-dimension input shapes shown in Figures 6A–D and compared the classification accuracy and #parameters when each shape was used as input. For comparison, the classification accuracy was also obtained for the case in which all 22 CHs of data were used with the channel configuration shown in Figure 6E. Note that zero was entered as the data point for the elements in the blank columns in Figure 6.

The classification accuracies were obtained under the same conditions and training environment as those in Table 1. The sample window size was set to 2.0 s, the #CHs was eight (22 was also included for comparison), and *n* = 2 for the subsample. The model parameters of the proposed 3D-CNN were set to *K*_s_ = 2, *K*_l_ = 16, *F*_1_ = 16, *D* = *2* (*F*_2_ = *F*_1_^*^ *D* = 32), Dropout was 0.5 for nine subjects, respectively. The same items of EEGNet were listed under these conditions. The classification accuracy of the proposed 3D-CNN with eight CHs was 0.7304–0.7360, which is 2.1%−2.7% better than that of EEGNet (0.7093), based on all eight CHs of EEGNet. However, because the classification accuracy varies depending on the combination of the model parameters, we provide details of the optimization of the model parameters in the next section. It should be noted that the classification accuracy did not change significantly with the shape of the input MI in the proposed 3D-CNN. The average classification accuracy was 0.7304 for the channel configuration (Figure 6A), in which the original electrode location was preserved as much as possible without removing unnecessary elements, whereas it was 0.7360, 0.7330, and 0.7321, for Figures 6B–D, in which the electrode location was changed from the actual location to compress the number of dimensions to avoid unnecessary calculations. The standard deviation was lowest for the proposed 3D-CNN (22 CHs), but there was no significant difference, ranging from 0.0148–0.0264. Additionally, the kappa score was nearly the same for all models (approximately 0.6133–0.6372), indicating that the stability of the models is almost identical. The fact that the classification accuracy can be maintained even if the dimensions of the input MI data are compressed by slightly changing the electrode locations is a great advantage in terms of reducing the number of parameters (#parameters) and the number of the multiply-accumulates (#MACCs). The #parameters of the input shapes in Figures 6B–D are 4.132 k, 3.812 k, and 3.812 k, respectively, which are 2.264–2.454 times smaller than those of EEGNet (eight CHs). #MACCs, Conv2D, and Depthwise Conv2D using a temporal filter in the first stage of EEGNet are integrated into one layer of Conv3D. Figures 6B–D are 0.63 M, 0.56 M, and 0.56 M, respectively, which are 1.730–1.946 times smaller than EEGNet (eight CHs). This was 48.6% lower than that of EEGNet (eight CHs) (1.09 M) at minimum. Similarly, the memory footprint was reduced by up to 63.9%. In contrast, when all 22 CHs of MI data were used in the proposed 3D-CNN and input into the model while maintaining the actual electrode locations, the classification accuracy was 0.7792, which was 4.72% better on average than that of EEGNet using all 22 CHs (0.7320). This suggests that the proposed 3D-CNN can increase classification accuracy by using a larger #channels and increasing the number of spatial features. Comparing Figures 6B–D, four out of nine subjects recorded the highest classification accuracy (bold in Table 6); therefore, the channel arrangement in Figure 6B was considered optimal and was used in subsequent analyses.

**Table 6:** Comparison of input MI shape, classification accuracy, number of parameters, computational complexity, and memory footprint of proposed 3D-CNN (EEGNet is also included for comparison) for BCI-IV2a.

**Model**		**EEGNet**		**Proposed 3D-CNN**				
#channels [CH]		8	22	8	8	8	8	22
Sample window size [s]		2.0	2.0	2.0	2.0	2.0	2.0	2.0
Subsample, *n*		2	2	2	2	2	2	2
Input data shape		(1, 8, *T*)	(1, 22, *T*)	Figure 6A (1, 6, 7, *T*)	Figure 6B **(1, 3, 3**, ***T*****)**	Figure 6C (1, 2, 4, *T*)	Figure 6D (1, 1, 8, *T*)	Figure 6E (1, 6, 7, *T*)
Model parameters		*K*_l_ = 64, *F*_1_ = 8, *D =* 2, *F*_2_ *=* 16, *K*_l2_ = 16, Dropout rate = 0.5		*K*_s_ = 2, *K*_l_ = 16, *F*_1_ = 16, *D =* 2 (*F*_2_ = *F*_1_^*^D = 32), Dropout rate = 0.5				
Accuracy	Sub1	0.7819	0.8049	0.8000	**0.8042**	0.7944	0.7958	0.8194
	Sub2	0.5472	0.5743	**0.5382**	0.5243	0.4979	0.5083	0.5923
	Sub3	0.8653	0.8681	0.8833	0.8792	**0.8854**	0.8938	0.9083
	Sub4	0.6257	0.6139	0.6778	0.6763	**0.6826**	0.6743	0.7285
	Sub5	0.6688	0.7507	0.6861	**0.7063**	0.6819	0.6986	0.7882
	Sub6	0.6125	0.5965	0.6340	**0.6604**	0.6576	0.6493	0.7034
	Sub7	0.7667	0.8694	0.8118	**0.8181**	0.8146	0.8146	0.8132
	Sub8	0.7785	0.7778	0.7757	0.7840	**0.7917**	0.7785	0.8299
	Sub9	0.7368	0.7326	0.7667	0.7715	**0.7910**	0.7757	0.8291
	Mean	0.7093	0.7320	0.7304	**0.7360**	0.7330	0.7321	0.7793
	{Difference: EEGNet (8CHs) = 0}	(0.000)	(0.023)	(0.021)	**(0.027)**	(0.024)	(0.023)	(0.070)
	Std. dev.	0.0264	0.0232	0.0171	0.0175	**0.0148**	0.0156	0.0171
Kappa score		0.6133	0.6384	0.6340	0.6394	0.6374	0.6372	0.6747
#parameters [k]		1.684	1.908	14.692	4.132	**3.812**	**3.812**	14.692
{Ratio: EEGNet(8CHs) = 1}		(1.000)	(1.133)	(8.724)	(2.454)	**(2.264)**	**(2.264)**	(8.724)
#MACCs [M]		1.09	2.94	2.87	0.63	**0.56**	**0.56**	2.87
{Ratio: EEGNet(8CHs) = 1}		(1.000)	(2.697)	(2.633)	(0.578)	**(0.514)**	**(1.028)**	(2.633)
Memory footprint [MB]		0.36	0.82	0.21	0.14	**0.13**	**0.13**	0.21
{Ratio: EEGNet(8CHs) = 1}		(1.000)	(2.278)	(0.583)	(0.389)	**(0.361)**	**(0.361)**	(0.583)

The best values (except for 22 CHs) are highlighted in bold.

### 3.3 Optimization of models' parameters for BCI-IV2a and BCI-IV2b

As mentioned in Section 3.2, in general, the various models' parameters that determine the size of deep-learning models (e.g., *K*_l_, *K*_l2_, *F*_1_*, D* for determining the model size in EEGNet) depend on the classification accuracy, computational complexity, and memory footprint. The optimal combination is expected to vary depending on the task performed. In this section, we seek the optimal values of the models' parameter combinations for BCI-IV2a and BCI-IV2b with a fixed #CHs for eight CHs (BCI-IV2a) and two CHs (BCI-IV2b), and a sample window size of 2.0 s (BCI-IV2a) and 3.0 s (BCI-IV2b), respectively. The subsample, *n* is set to 1 and 2 for both BCI-IV2a and BCI-IV2b. The combinations of model parameters for the five models [EEGNet (Lawhern et al., 2018), EEG-TCNet (Ingolfsson et al., 2020), TCNet-Fusion (Musallam et al., 2021), EISATC-Fusion (Liang et al., 2024), and the proposed-3D CNN] are as follows (default settings in bold):

EEGNet {Dropout (*p*) is fixed at 0.5}

- *K*_l_ {**64**, 32, 16, 8, 4, 2, 1}, *F*_1_ {**8**, 4, 2, 1}, *F*_2_ (***F**_1_* ^*^ ***D***), *D*{**2**}, *K*_l2_ {**16**, 8, 4, 2, 1}

EEG-TCNet {Dropout (*p*_*e*_, *p*_*t*_) is fixed at 0.3}

- *F*_1_ {**8**, 4, 2, 1}, *F*_2_ {***F**_1_* ^*^ ***D***}, *D* {**2**}, *K*_*E*_{**32**, 16, 8, 4, 2, 1}, *K*_*T*_{**4**, 2, 1}, *L* {**2**}, *F*_*T*_{**12**, 4, 2, 1}

TCNet-Fusion {Dropout (*p*_*e*_, *p*_*t*_) is fixed at 0.3}

- *F*_1_ {**24**, 12, 8, 4, 2, 1}, *F*_2_ {***F**_1_* ^*^ ***D***}, *D* {**2**}, *K*_*E*_{**32**, 16, 8, 4, 2, 1}, *K*_*T*_{**4**, 2, 1}, *L* {**2**}, *F*_*T*_{**12**, 4, 2, 1}

EISATC-Fusion{Dropout (*p*_*e*_, *p*_*t*_) is fixed at 0.3}

- *F*_1_ {**16**, 8, 4, 2, 1}, *F*_2_ {***F**_1_* ^*^ ***D***}, *D* {**2**}, *K*_*E*_{**32**, 16, 8, 4, 2, 1}, *K*_*T*_{**4**, 2, 1}, *L* {**2**}, *F*_*T*_ {**32**, 16, 8, 4, 2, 1},

Proposed 3D-CNN {Dropout (*p*) is fixed at 0.5}

- *K*_s_ {3, **2**, 1}, *K*_l_ {**16**, 12, 8, 4, 2, 1}, *F*_1_ {**16**, 8, 4, 2, 1}, *D* {**2**}, *F*_2_ {***F**_1_* ^*^ ***D***}

**Figure 7:**
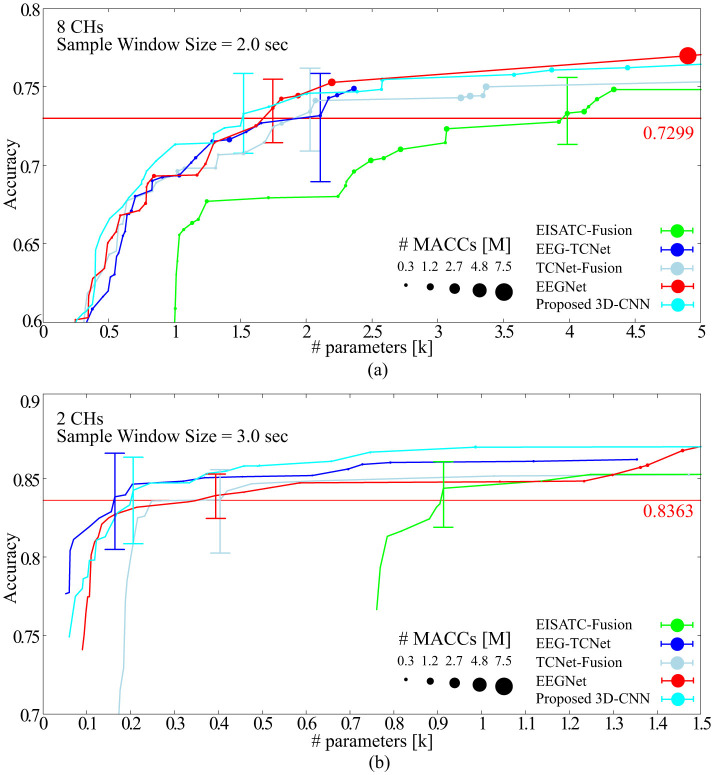
Pareto-front (Classification accuracy vs. #parameters of models) **(A)** BCI-IV2a **(B)** BCI-IV2b.

Training was performed on BCI-IV2a under the aforementioned conditions using the measurement conditions of 8CHs, 2 s, and subsampling *n* = 1, 2, 3, 4, and 5. Figure 7A shows the Pareto front (average classification accuracy vs. #parameters). The Pareto front plots the maximum mean classification accuracy achieved using the #parameters. The relationship between the maximum classification accuracy that can be achieved under the given conditions and the #parameters can be visualized by sorting the #parameters of the target model in ascending order and plotting only when the maximum classification accuracy is updated. Therefore, the higher the curve ascends, the greater the classification accuracy achieved with fewer parameters. Among the five models considered, the proposed 3D-CNN, EEGNet, EEG-TCNet, and TCNet-Fusion were nearly positioned the same, with EISATC-Fusion slightly farther to the right. Additionally, when the number of parameters are limited to 1 k or fewer, the proposed 3D-CNN (light blue) is positioned along the top-left line, indicating that it achieves the highest classification accuracy with fewer parameters. The solid red line represents 0.7299, the “minimum classification accuracy of 5-fold cross-validation using the largest 22 CHs with a 4.0 s sample window” in EEGNet, as described in Section 2.2.3. For visualization, the error bar is shown only for the smallest #parameters where the average classification accuracy exceeds 0.7299. Table 7 lists the values of each model parameter, classification accuracies for each subject, #parameters, #MACCs, and memory footprint at this error bar. As the classification accuracies in this table are similar, they were not compared. The standard deviation ranges from 0.0154 to 0.0249, indicating no significant variation between the five models. The kappa scores are also similar, ranging from 0.6389–0.6416. In contrast, the proposed 3D-CNN has the lowest #parameters at 1.524 k, approximately 13% lower than EEGNet. The order of #parameters is EEGNet (1.748 k), TCNet-Fusion (2.03 k), EEG-TCNet (2.108 k), and EISATC-Fusion (3.985 k). Additionally, the proposed 3D-CNN has the lowest #MACCs at 0.17 M, about 56% lower than EEGNet. The other values are in the following order: EEG-TCNet (0.36 M), EEGNet (0.39 M), TCNet-Fusion (0.61 M), and EISATC-Fusion (0.61 M). As shown in the table, the proposed 3D-CNN has the smallest memory footprint of 0.14 MB, approximately 80% less than EEGNet. The order of memory footprints is as follows: EEG-TCNet (0.69 MB), TCNet-Fusion (0.69 MB), EISATC-Fusion (0.70 MB), and EEGNet (0.71 MB). The lines for the proposed 3D-CNN and EEGNet reverse at around 2 k #parameters; however, #MACCs (plot size) is larger for EEGNet than for the proposed 3D-CNN. Therefore, the proposed 3D-CNN improves average classification accuracy while limiting the increase in #MACCs.

**Table 7:** Comparison of number of parameters, computational complexity, and memory footprint at the same level of classification accuracy of the five models for BCI-IV2a.

**Model**		**EEGNet (Lawhern et al., 2018)**	**EEG-TCNet (Ingolfsson et al., 2020)**	**TCNet-Fusion (Musallam et al., 2021)**	**EISATC-Fusion (Liang et al., 2024)**	**Proposed 3D-CNN**
#channels [CH]		8	8	8	8	8
Sample window size [s]		2.0	2.0	2.0	2.0	2.0
Subsample, *n*		1	1	1	1	1
Input data shape		(1, 8, *T*)	(1, 8, *T*)	(1, 8, *T*)	(1, 8, *T*)	Figure 6B (1, 3, 3, *T*)
Model parameters		*K*_l_ = 16, *F*_1_ = 8, *D* = 2, *F*_2_ = 16, *K*_l2_ = 8, Dropout rate = 0.5	*F*_1_ = 8, *D* = 2, *F*_T_ = 4, *F*_2_ = 16, *K_*E*_* = 8, *K_*T*_* = 4, Dropout rate (*p_*e*_, p_*t*_*) = 0.3	*F*_1_ = 8, *D* = 2, *F*_T_ = 16, *F*_2_ = 16, *K_*E*_* = 2, *K_*T*_* = 4, Dropout rate (*p_*e*_, p_*t*_*) = 0.3	*F*_1_ = 8, *D* = 2, *F*_2_ = 16, *F_*T*_* = 16, *K_*E*_* = 4, *K_*T*_* = 4, Dropout rate(*p_*e*_, p_*t*_*) = 0.3	*K*_s_ = 1, *K*_l_ = 4, *F*_1_ = 8, *D* = 2, *F*_2_ = 16, Dropout rate = 0.5
Accuracy	Sub1	0.7785	0.7785	0.7854	0.7979	0.7792
	Sub2	0.6188	0.6111	0.5576	0.6028	0.5632
	Sub3	0.8944	0.8451	0.8833	0.8819	0.8813
	Sub4	0.6694	0.6931	0.6972	0.7035	0.6715
	Sub5	0.6972	0.6847	0.7090	0.6757	0.7056
	Sub6	0.6451	0.6389	0.6222	0.5674	0.6417
	Sub7	0.7924	0.8069	0.8035	0.8076	0.8000
	Sub8	0.7951	0.7674	0.8056	0.7674	0.7889
	Sub9	0.7389	0.7597	0.7438	0.7951	0.7646
	Mean	0.7367	0.7317	0.7342	0.7333	0.7329
	(Difference: EEGNet = 0)	(0.0000)	(−0.0049)	(−0.0025)	(−0.0034)	(−0.0038)
	Std. dev.	**0.0154**	0.0249	0.0196	0.0160	0.0185
Kappa score		0.6416	0.6389	0.6408	0.6401	0.6396
#parameters [k]		1.748	2.108	2.03	3.985	**1.524**
(Ratio: EEGNet = 1)		(1.0000)	(1.2059)	(1.1613)	(2.2797)	**(0.8719)**
#MACCs [M]		0.39	0.36	0.61	0.61	**0.17**
(Ratio: EEGNet = 1)		(1.0000)	(0.9231)	(1.5641)	(1.5641)	**(0.4359)**
Memory footprint [MB]		0.71	0.69	0.69	0.7	**0.14**
(Ratio: EEGNet = 1)		(1.0000)	(0.9718)	(0.9718)	(0.9859)	**(0.1972)**

The best values are highlighted in bold.

Training was conducted on BCI-IV2b, similar to BCI-IV2a, using measurement conditions of 2 CHs, 3 s, and subsampling *n* = 1, 2, 3, 4, and 5. Figure 7B shows the Pareto front (average classification accuracy vs. #parameters). Among the five models, the proposed 3D-CNN, EEGNet, EEG-TCNet, and TCNet-Fusion were almost at the same position, with EISATC-Fusion slightly farther to the right. When #parameters is limited to 0.4 k or less, EEG-TCNet (blue) appears at the top left, indicating it achieves the highest classification accuracy with smaller #parameters. Conversely, when #parameters exceed 0.4 k, the proposed 3D-CNN (light blue) achieves the highest classification accuracy. The solid red line represents 0.8363, the “minimum classification accuracy from 5-fold cross-validation using the largest 3 CHs with a 4.0 s sample window size” in EEGNet, as described in Section 2.2.6. For visualization, the error bar is shown only for the smallest #parameters where the average classification accuracy exceeds the red target line (0.8363). Table 8 lists the values for each model parameter, classification accuracy for each subject, #parameters, #MACCs, and memory footprint at this error bar. As the classification accuracies in this table are similar, they were not compared. As observed from the table, the standard deviation ranges from 0.0108 to 0.0220, indicating no significant variation between the five models. Additionally, the kappa scores are similar, ranging from 0.6652–0.6823. In contrast, #parameters is lowest for EEG-TCNet at 0.164 k, approximately 58% lower than EEGNet. The order of #parameters is as follows: proposed 3D-CNN (0.206 k), EEGNet (0.394 k), TCNet-Fusion (0.404 k), and EISATC-Fusion (0.913 k). Moreover, EEG-TCNet had the lowest #MACCs at 0.02 M, approximately 50% lower than EEGNet. The #MACCs of the proposed 3D-CNN (0.03 M) and TCNet-Fusion (0.03 M) are similar to EEG-TCNet.

**Table 8:** Comparison of number of parameters, computational complexity, and memory footprint at the same level of classification accuracy of the five models for BCI-IV2b.

**Model**		**EEGNet (Lawhern et al., 2018)**	**EEG-TCNet (Ingolfsson et al., 2020)**	**TCNet-Fusion (Musallam et al., 2021)**	**EISATC-Fusion (Liang et al., 2024)**	**Proposed 3D-CNN**
#channels [CH]		2	2	2	2	2
Sample window size [s]		3.0	3.0	3.0	3.0	3.0
Subsample, *n*		4	2	1	1	3
Input data shape		(1, 2, *T*)	(1, 2, *T*)	(1, 2, *T*)	(1, 2, *T*)	(1, 1, 2, *T*)
Model parameters		*K*_l_ = 16, *F*_1_ = 4, *D* = 2, *F*_2_ = 8, *K*_l2_ = 16, Dropout rate = 0.5	*F*_1_ = 8, *D* = 2, *F*_T_ = 8, *F*_2_ = 16, *K_*E*_* = 2, *K_*T*_* = 2, Dropout rate (*p_*e*_, p_*t*_*) = 0.3	*F*_1_ = 2, *D* = 2, *F*_T_ = 4, *F*_2_ = 4, *K_*E*_* = 2, *K_*T*_* = 4, Dropout rate (*p_*e*_, p_*t*_*) = 0.3	*F*_1_ = 4, *D* = 2, *F*_2_ = 8, *F_*T*_* = 4, *K_*E*_* = 2, *K_*T*_* = 2, Dropout rate (*p_*e*_, p_*t*_*) = 0.3	*K*_s_ = 2, *K*_l_ = 2, *F*_1_ = 16, *D* = 2, *F*_2_ = 32, Dropout rate = 0.5
Accuracy	Sub1	0.7050	0.6994	0.7119	0.7044	0.7006
	Sub2	0.6671	0.7057	0.7257	0.7193	0.6729
	Sub3	0.8744	0.8769	0.8581	0.8819	0.8619
	Sub4	0.9613	0.9325	0.9350	0.9431	0.9619
	Sub5	0.9494	0.9313	0.9606	0.9581	0.9181
	Sub6	0.7400	0.7669	0.7631	0.7756	0.7969
	Sub7	0.8919	0.9006	0.9075	0.9188	0.8481
	Sub8	0.9481	0.9363	0.9250	0.9250	0.9406
	Sub9	0.8163	0.7963	0.7419	0.7688	0.8825
	Mean	0.8393	0.8384	0.8365	0.8439	0.8426
	(Difference: EEGNet = 0)	(0.0000)	(-0.0009)	(-0.0027)	(+0.0046)	(+0.0033)
	Std. dev.	**0.0108**	0.0220	0.0195	0.0156	0.0200
Kappa score		0.6813	0.6788	0.6652	0.6823	0.6811
#parameters [k]		0.394	**0.164**	0.404	0.913	0.206
(Ratio: EEGNet = 1)		(1.0000)	**(0.4162)**	(1.0254)	(2.3173)	(0.5228)
#MACCs [M]		0.04	**0.02**	0.03	0.05	0.03
(Ratio: EEGNet = 1)		(1.0000)	**(0.5000)**	(0.7500)	(1.2500)	(0.7500)
Memory footprint [MB]		0.06	0.06	0.11	0.22	**0.02**
(Ratio: EEGNet = 1)		(1.0000)	(1.0000)	(1.8333)	(3.6667)	**(0.3333)**

The best values are highlighted in bold.

The order for the other values was EEGNet (0.04 M) and EISATC-Fusion (0.05 M). The proposed 3D-CNN had the smallest memory footprint of 0.02 MB, approximately 66% less than that of EEGNet. The order of memory footprints was EEGNet (0.06 MB), EEG-TCNet (0.06 MB), TCNet-Fusion (0.11 MB), and EISATC-Fusion (0.22 MB). This aligns with the case of BCI-IV2a, where the proposed 3D-CNN improved mean classification accuracy while suppressing increases in #MACCs and memory footprint in the lower-class dataset (BCI-IV2b).

## 4 Motor imagery measurement demonstration

In the previous section, we used the BCI Competition IV-2a Dataset (BCI-IV2a) and the BCI Competition IV-2b Dataset (BCI-IV2b), international benchmarks for MI using EEG measurements, to estimate the optimal values for the electrode channels (CHs), sample window size, and number of subsamples (*n*) based on the classification accuracy in MI measurements. In this section, in order to demonstrate the effectiveness of these methods and reduce the burden on users, we obtained new data for Dry MI (MI with dry electrodes), following the method on BCI-IV2a. However, because the contact impedance between the dry electrode and the scalp is higher than that of the wet electrode using a conductive gel, the noise component of MI increases, and it is expected to be more difficult to reveal the feature components. Therefore, in this study, in addition to the usual instructions to the subject using still images, we also used moving images to reveal a larger number of features and obtained Dry-MI data for comparison.

### 4.1 Dry electrode to reduce user load, acquisition of new motor imagery EEG data using moving image materials for feature manifestation

**Figure 8:**
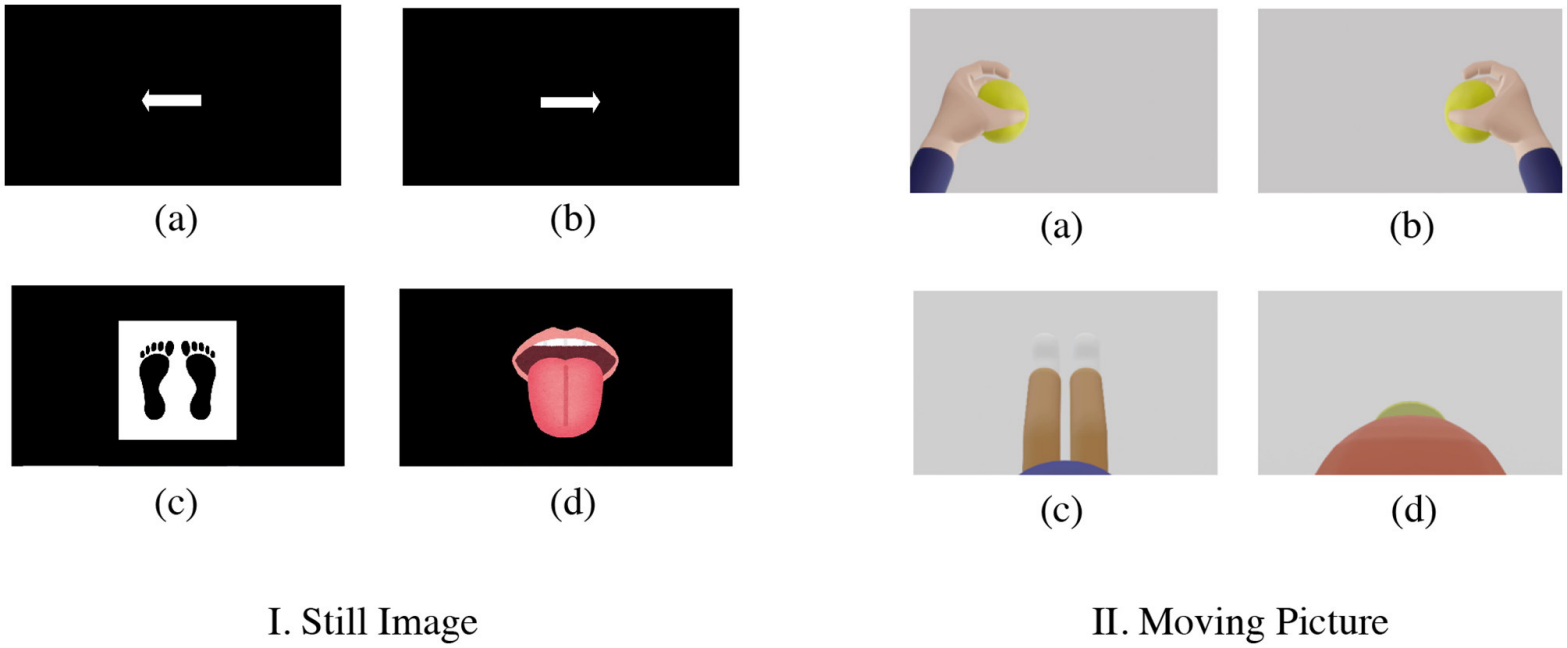
Presentation of cue by still image and moving picture [**I**. still image and **II**. moving picture (new MI-EEG acquisition) (a) left hand (b) right hand (c) both legs (d) tongue]. The following is a description of the motion indicated in each video: (a) Left hand: Gripping the ball slowly (b) Right hand: Same exercise as left hand (c) Legs: Bending and stretching exercises of the knees while sitting on a chair (d) Tongue: Exercise to lick candy.

Two subjects (both 22-years old, male), SubA and SubB, newly acquired MI using a g.Nautilus EEG system manufactured by g.tec and g.SAHARA dry active electrode system manufactured by g.tec, which is a dry active electrode. Both subjects acquired new MI using the g.SAHARA dry active electrode system. None of the participants had any known neurological disorders or serious health problems. The electrodes were placed at the red-filled locations in Figure 1 (C5, C3, CZ, C2, CP1, CP2, CP4, and POZ). The sampling frequency was 250 Hz, which is the same as that of BCI-IV2a. The timing paradigm was slightly different from that of BCI-IV2a (Figure 2): Fixation Cross (2 s), Cue (1.25 s), Motor Imagery (4 s), and Break (2 s); however, the flow was generally the same. During the Cue segment, the participants were presented with a still image (Figure 8I) and a moving image (Figure 8II). To ensure that all experimental conditions were the same, all BCI-IV2a acquisition conditions (number of sessions, runs, and trials) were the same, and each session was conducted on a different day. In two sessions, a still image was presented as a cue. In the other two sessions, a moving image was presented as a cue.

### 4.2 Sample window size dependency

**Figure 9:**
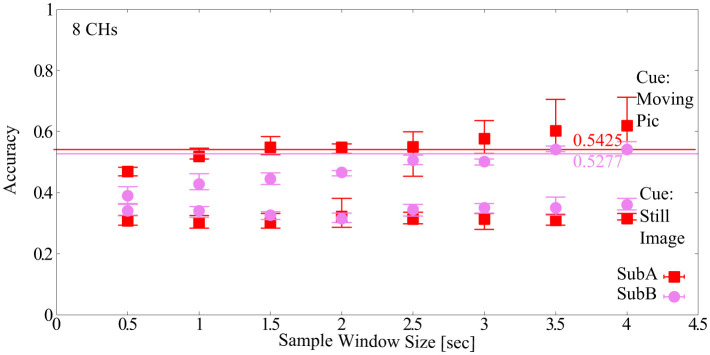
Classification accuracy vs. sample window size for newly acquired Dry-MI.

The sample window size dependence of the newly acquired Dry-MI was confirmed. The results are shown in Figure 9. The number of channels (#CHs) was 8 CHs and the default model parameters of EEGNet were used. First, when the cue was a still image, the classification accuracy was approximately 30% for both subjects (SubA and SubB) at all sample window sizes, which is considerably low compared to the chance level of 25% (4-class). This may be due to the fact that the feature values are not dependent on the sample window size or subjects, and that there is no correlation between session 1 (training Dry-MI) and session 2 (test Dry-MI). On the other hand, when the cue was changed to a moving picture, the mean classification accuracy was approximately 60% (sample window size = 4.0 s), although there was some variation between the two subjects. Compared to the BCI-IV2a, the Dry-MI showed a larger increase or decrease in classification accuracy depending on the sample window size. The maximum sample window size that can maintain the classification accuracy was determined to be the minimum classification accuracy of 5-fold cross validation obtained with sample window size = 4.0 s as in Section 2.2.3 (SubA = 0.5425, SubB = 0. 5277). However, in Section 2.2.3, the classification accuracy was based on 22 CHs, but since there is no corresponding data for Dry-MI, 8 CHs is used here. As a result, the optimal sample window size for the two subjects was 3.5 s. However, it should be noted that this is the result of two subjects and not the average of many subjects.

**Figure 10:**
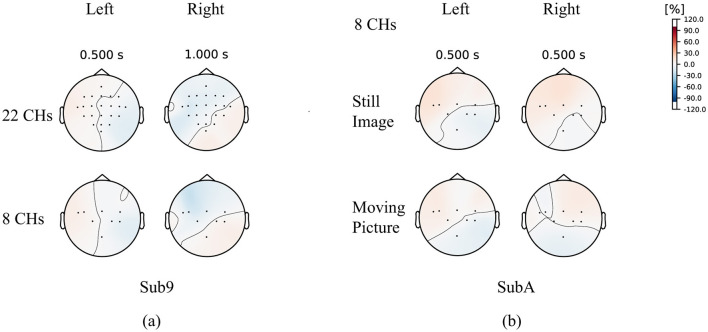
ERD/ERS topography **(A)** Sub9, BCI-IV2a **(B)** SubA, newly acquired Dry-MI.

To investigate the difference between wet MI (Wet-MI) and dry MI (Dry-MI), the event-related desynchronization and synchronization (ERD/ERS) (Rimbert et al., 2023) topography for Wet-MI during “left” and “right” recall in Sub9 of BCI-IV2a is shown as an example in Figure 10A. Desynchronization occurred around the motor cortex and was stronger on the side opposite the recall direction. This feature was observed even when #CHs was reduced from 22 to 8, suggesting it contributed to the minimal decrease in classification accuracy. Figure 10B shows the topography for “left” and “right” recall in SubA, representing Dry-MI. In the case of the Still Image, strong desynchronization occurred in the same direction for both the “left” and “right” cases, which is considered a factor in the difficulty of classification due to the similarity of the features. However, in the case of the Moving Picture, desynchronization in the opposite direction was less pronounced than in BCI-IV2a, though a certain difference was still observed, which is thought to improve classification accuracy.

### 4.3 Model parameter dependency

**Figure 11:**
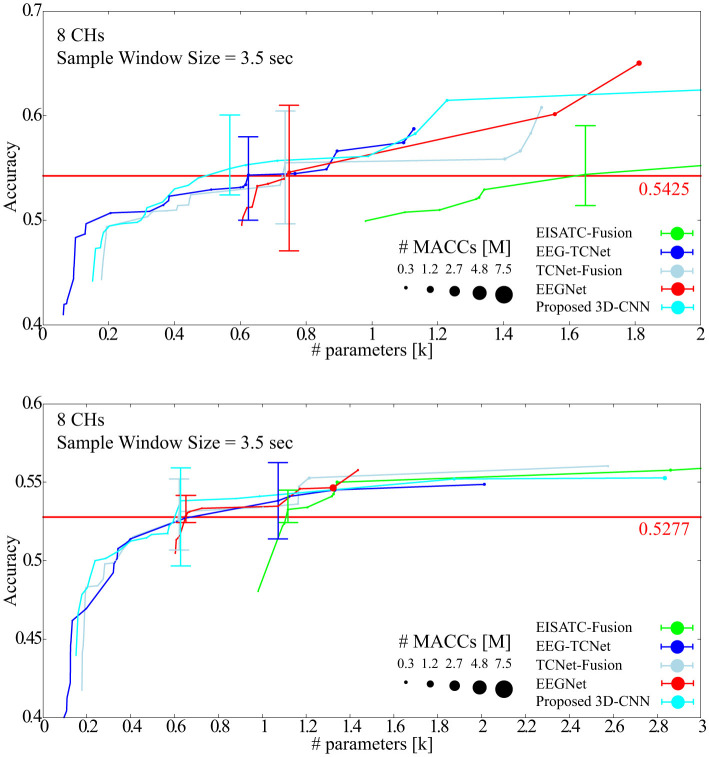
Pareto-front (Classification accuracy vs. #parameters of models) **(A)** SubA **(B)** SubB.

In this section, the optimal model parameters for the new Dry-MI were obtained in the same way as in Section 3.3. Figure 11A (SubA) and Figure 11B (SubB) shows the Pareto front (average classification accuracy vs. #parameters). As in Section 4.2, the minimum number of model parameters (#parameters) that can maintain the minimum classification accuracy (SubA = 0.5425, SubB = 0.5277) of the 5-fold cross validation obtained with the sample window size = 4.0 s is defined as error bar in Figure 11. Table 9 (SubA) and Table 10 (SubB) show the model conditions in this experiment.

**Table 9:** Comparison of number of parameters, computational complexity, and memory footprint at the same level of classification accuracy among 5 deep-learning models for newly measured motor imagery (SubA).

**Model**		**EEGNet (Lawhern et al., 2018)**	**EEG-TCNet (Ingolfsson et al., 2020)**	**TCNet-Fusion (Musallam et al., 2021)**	**EISATC-Fusion (Liang et al., 2024)**	**Proposed 3D-CNN**
#channels [CH]		8	8	8	8	8
Sample window size [s]		3.5	3.5	3.5	3.5	3.5
Subsample, *n*		2	1	2	2	2
Input data shape		(1, 8, *T*)	(1, 8, *T*)	(1, 8, *T*)	(1, 8, *T*)	Figure 6B (1, 3, 3, *T*)
Model parameters		*K*_l_ = 32, *F*_1_ = 4, *D* = 2, *F*_2_ = 8, *K*_l2_ = 4, Dropout rate = 0.5	*F*_1_ = 4, *D* = 2, *F*_T_ = 2, *F*_2_ = 8, *K_*E*_* = 4, *K_*T*_* = 4, Dropout rate (*p_*e*_, p_*t*_*) = 0.3	*F*_1_ = 4, *D* = 2, *F*_T_ = 8, *F*_2_ = 8, *K_*E*_* = 1, *K_*T*_* = 4, Dropout rate (*p_*e*_, p_*t*_*) = 0.3	*F*_1_ = 4, *D* = 2, *F*_2_ = 8, *F_*T*_* = 8, *K_*E*_* = 4, *K_*T*_* = 2, Dropout rate (*p_*e*_, p_*t*_*) = 0.3	*K*_s_ = 3, *K*_l_ = 8, *F*_1_ = 2, *D* = 2, *F*_2_ = 4, Dropout rate = 0.5
Accuracy	SubA	0.5458	0.5431	0.5549	0.5438	0.5493
	(Difference: EEGNet = 0)	(0.0000)	(-0.0028)	(+0.0090)	(-0.0021)	(0.0035)
	Std. dev.	0.0606	0.0322	0.0371	0.0286	**0.0277**
Kappa score		0.4011	0.4003	0.4126	0.4009	0.4063
#parameters [k]		0.748	0.624	0.737	1.649	**0.568**
(Ratio: EEGNet = 1)		(1.0000)	(0.8342)	(0.9853)	(2.2045)	**(0.7594)**
#MACCs [M]		0.49	0.29	0.15	0.16	**0.08**
(Ratio: EEGNet = 1)		(1.0000)	(0.5918)	(0.3061)	(0.3265)	**(0.1633)**
Memory footprint [MB]		0.32	0.62	0.31	0.31	**0.04**
(Ratio: EEGNet = 1)		(1.0000)	(1.9375)	(0.9688)	(0.9688)	**(0.1250)**

The best values are highlighted in bold.

**Table 10:** Comparison of number of parameters, computational complexity, and memory footprint at the same level of classification accuracy among 5 deep-learning models for newly measured motor imagery (SubB).

**Model**		**EEGNet (Lawhern et al., 2018)**	**EEG-TCNet (Ingolfsson et al., 2020)**	**TCNet-Fusion (Musallam et al., 2021)**	**EISATC-Fusion (Liang et al., 2024)**	**Proposed 3D-CNN**
#channels [CH]		8	8	8	8	8
Sample window size [s]		3.5	3.5	3.5	3.5	3.5
Subsample, *n*		2	1	1	2	2
Input data shape		(1, 8, *T*)	(1, 8, *T*)	(1, 8, *T*)	(1, 8, *T*)	Figure 6B (1, 3, 3, *T*)
Model parameters		*K*_l_ = 8, *F*_1_ = 4, *D* = 2, *F*_2_ = 8, *K*_l2_ = 4, Dropout rate = 0.5	*F*_1_ = 8, *D* = 2, *F*_T_ = 8, *F*_2_ = 16, *K_*E*_* = 4, *K_*T*_* = 4, Dropout rate (*p_*e*_, p_*t*_*) = 0.3	*F*_1_ = 2, *D* = 2, *F*_T_ = 8, *F*_2_ = 4, *K_*E*_* = 1, *K_*T*_* = 4, Dropout rate (*p_*e*_, p_*t*_*) = 0.3	*F*_1_ = 4, *D* = 2, *F*_2_ = 8, *F_*T*_* = 8, *K_*E*_* = 2, *K_*T*_* = 2, Dropout rate (*p_*e*_, p_*t*_*) = 0.3	*K*_s_ = 1, *K*_l_ = 4, *F*_1_ = 4, *D* = 2, *F*_2_ = 8, Dropout rate = 0.5
Accuracy	SubB	0.5285	0.5382	0.5313	0.5326	0.5382
	(Difference: EEGNet = 0)	(0.0000)	(0.0097)	(0.0028)	(0.0042)	(0.0097)
	Std. dev.	**0.0067**	0.0173	0.0182	0.0081	0.0219
Kappa score		0.3713	0.3825	0.3727	0.3795	0.3842
#parameters [k]		0.652	1.072	**0.621**	1.117	0.628
(Ratio: EEGNet = 1)		(1.0000)	(1.6442)	**(0.9525)**	(1.7132)	(0.9632)
#MACCs [M]		0.15	0.21	0.09	0.16	**0.04**
(Ratio: EEGNet = 1)		(1.0000)	(1.4000)	(0.6000)	(1.0667)	**(0.2667)**
Memory footprint [MB]		0.32	1.2	0.32	0.31	**0.07**
(Ratio: EEGNet = 1)		(1.0000)	(3.7500)	(1.0000)	(0.9688)	**(0.2188)**

The best values are highlighted in bold.

SubA (Figure 11A) illustrates the same trend observed in BCI-IV2a (Figure 7A). Among the five models considered, the proposed 3D-CNN, EEGNet, EEG-TCNet, and TCNet-Fusion were nearly identical, with EISATC-Fusion slightly farther to the right. For visualization, the error bar is shown only for the smallest #parameters where the average classification accuracy exceeds 0.5425. Table 9 shows that while there is no significant difference in standard deviations and kappa scores among the models, the proposed 3D-CNN has the smallest model size. It also achieves the lowest #parameters (approximately 25% smaller than EEGNet), #MACCs (approximately 83% smaller than EEGNet), and memory footprint (approximately 87% smaller than EEGNet). SubB (Table 10) follows a similar trend, with the proposed 3D-CNN achieving the same classification accuracy as TCNet-Fusion but with the second smallest #parameters (0.628k, compared to TCNet-Fusion's 0.621k). Its #MACCs (approximately 83% less than EEGNet) and memory footprint (approximately 78% less than EEGNet) were also the smallest among the five models. These results suggest that the proposed 3D-CNN is an effective model for maintaining MI classification accuracy for both Wet-MI and Dry-MI while reducing model size.

## 5 Conclusion

In this study, we focused on motor imagery (MI), which requires only recall and not a stimulus presentation device, with the goal of utilizing the BMI in living spaces to address social challenges in nursing care and aimed to simplify the MI-BMI system. The MI-BMI system can operate in real-time on a battery-powered edge device with limited computational resources for extended periods by reducing the size of the deep-learning model to classify MI, achieving high MI classification accuracy while minimizing computation and memory usage. By implementing an edge-based BMI system, privacy is ensured. We optimized the MI measurement conditions by reducing the number of channels (#CHs), optimizing channel positions, shortening the recall time, and lowering the sampling frequency to simplify the BMI system. Additionally, to maintain classification accuracy, a 3D-CNN was used to minimize the parameter size of the model by incorporating channel placement information (spatial data) into the input. We also reduced the burden on the user by using dry electrodes and compared the effectiveness of the proposed 3D-CNN with EEGNet, EEG-TCNet, TCNet-Fusion, and EISATC-Fusion as deep-learning models. We acquired eight-channel dry-MI data from two subjects. Compared to EEGNet, the proposed 3D-CNN reduces #parameters, #MACCs, and memory footprint by approximately 75.9%, 16.3%, and 12.5%, respectively, while maintaining the same level of classification accuracy. However, this optimization was limited to five deep-learning models and within-subject MI decoding. In the future, it will be necessary to optimize other deep-learning models to identify the optimal model for edge implementation. Additionally, the dry-MI used in this study was a trial version with only two subjects. A larger number of subjects is needed, along with optimization of cross-subject MI decoding. Future research will focus on developing experimental strategies to obtain clearer MI features when using dry electrodes. This study lays the foundation for this by demonstrating the use of moving pictures as a cue in the experiment.

## Data Availability

The datasets presented in this article are not readily available because the generated datasets are prohibited to share in a publicly accessible repository yet. Requests to access the datasets should be directed to Nobuaki Kobayashi, kobayashi.nobuaki@nihon-u.ac.jp.
